# Engineering Thermoresponsive Poly(*N*-isopropylacrylamide)-Based Films with Enhanced Stability and Reusability for Efficient Bone Marrow Mesenchymal Stem Cell Culture and Harvesting

**DOI:** 10.3390/molecules29184481

**Published:** 2024-09-21

**Authors:** Lei Yang, Luqiao Sun, Yuanyuan Sun, Guangwei Qiu, Xiaoguang Fan, Qing Sun, Guang Lu

**Affiliations:** 1 School of Petrochemical Engineering, Liaoning Petrochemical University, Fushun 113001, China; yanglei@lnpu.edu.cn (L.Y.); sunluqiao@stu.lnpu.edu.cn (L.S.); qiuguangwei@stu.lnpu.edu.cn (G.Q.); 2 College of Engineering, Shenyang Agricultural University, Shenyang 110866, China; 2022240009@stu.syau.edu.cn; 3 School of Civil Engineering, Liaoning Petrochemical University, Fushun 113001, China; luguang@lnpu.edu.cn

**Keywords:** poly(*N*-isopropylacrylamide) (PNIPAM), bone marrow mesenchymal stem cells (BMMSCs), cell culture and harvest, film stability and reusability

## Abstract

Poly(*N*-isopropylacrylamide) (PNIPAM) offers a promising platform for non-invasive and gentle cell detachment. However, conventional PNIPAM-based substrates often suffer from limitations including limited stability and reduced reusability, which hinder their widespread adoption in biomedical applications. In this study, PNIPAM copolymer films were formed on the surfaces of glass slides or silicon wafers using a two-step film-forming method involving coating and grafting. Subsequently, a comprehensive analysis of the films’ surface wettability, topography, and thickness was conducted using a variety of techniques, including contact angle analysis, atomic force microscopy (AFM), and ellipsometric measurements. Bone marrow mesenchymal stem cells (BMMSCs) were then seeded onto PNIPAM copolymer films prepared from different copolymer solution concentrations, ranging from 0.2 to 10 mg·mL^−1^, to select the optimal culture substrate that allowed for good cell growth at 37 °C and effective cell detachment through temperature reduction. Furthermore, the stability and reusability of the optimal copolymer films were assessed. Finally, AFM and X-ray photoelectron spectroscopy (XPS) were employed to examine the surface morphology and elemental composition of the copolymer films after two rounds of BMMSC adhesion and detachment. The findings revealed that the surface properties and overall characteristics of PNIPAM copolymer films varied significantly with the solution concentration. Based on the selection criteria, the copolymer films derived from 1 mg·mL^−1^ solution were identified as the optimal culture substrates for BMMSCs. After two rounds of cellular adhesion and detachment, some proteins remained on the film surfaces, acting as a foundation for subsequent cellular re-adhesion and growth, thereby implicitly corroborating the practicability and reusability of the copolymer films. This study not only introduces a stable and efficient platform for stem cell culture and harvesting but also represents a significant advance in the fabrication of smart materials tailored for biomedical applications.

## 1. Introduction

Stem cells, possessing both self-renewal and multipotentiality for differentiation, have the ability to repair diseased cells or rebuild healthy ones in patients after transplantation [1,2]. Their therapeutic value is ascribable to their low (or non-existent) toxicity, broad therapeutic range, non-rejection from the immune system, and noteworthy curative effect. However, a major obstacle lies in the scarcity of stem cells derived from human or animal tissues and fluids, making it challenging to satisfy clinical needs [3,4]. Thus, developing an efficient in vitro stem cell amplification system becomes essential. During in vitro culture, two pivotal aspects must be addressed, enhancing stem cell proliferation while preserving their unique properties. This is fundamental for ensuring a reliable supply of stem cells in biomedical applications.

Adherent stem cells encounter repetitive inoculation and harvesting cycles during in vitro cultivation. A tailored culture substrate, optimal culture conditions, and an apt cell harvesting technique are imperative for yielding ample quantities of high-quality stem cells in the realm of tissue engineering. Currently, enzymatic digestion stands as the prevalent cell harvesting method, which relies on selectively breaking down protein peptide chains to disentangle the adhesion between cells and the substrate, forcing cell to detach [5,6,7]. Nonetheless, enzyme introduction inflicts damage on cytomembrane proteins. While cellular self-repair mechanisms can temporarily compensate for dysfunction stemming from membrane surface protein impairments, prolonged in vitro cultivation or frequent passages can induce spontaneous differentiation or premature cell senescence, hindering the therapeutic potential of stem cells. Consequently, minimizing the reliance on enzymatic digestion or exploring alternative harvesting techniques is essential.

The advancement of thermoresponsive poly(*N*-isopropylacrylamide) (PNIPAM) has provided fresh perspectives in addressing the challenges. It stands as an efficient alternative to conventional enzymolysis techniques. PNIPAM is a temperature-sensitive polymer, exhibiting distinct overall configuration and surface wettability in response to temperature variations [8,9]. Adherent cells exhibit selectivity towards the surface characteristics of materials, and most of adhering cells demonstrate a preference for mildly hydrophobic surfaces and display an aversion towards strong hydrophilic surfaces [10,11]. Scientists have integrated the thermoresponsivity of PNIPAM-based materials with cellular preferences for culture substrate surfaces. By incorporating diverse functional groups or cytokines into the materials, they have created weakly hydrophobic surfaces that foster the attachment of varying cell types above the lower critical solution temperature (LCST) and hydrophilic surfaces that facilitate the detachment of cells below the LCST, pioneering a novel culture and recovery platform. By solely employing cooling treatment, PNIPAM-based substrates undergo a transition from hydrophobic to hydrophilic, resulting in the spontaneous detachment of adherent cells without the need for external enzymatic or mechanical intervention. This method of cell recovery through temperature reduction, also known as cooling-induced cell detachment method, enables the detachment of cells along with extracellular matrix proteins, safeguarding cytomembrane proteins and related receptors, thus sustaining cellular physiological functions [12,13,14]. In contrast to traditional enzymatic and mechanical techniques, this simple thermoresponsive material-based detachment approach preserves superior growth characteristics of cells. Okano’s group pioneered the utilization of PNIPAM in cell culture and non-enzymatic harvest, initiating a novel research trajectory in tissue repair and regenerative medicine, coined as “cell sheet engineering” [15,16]. This represents the dawn of a new era in the application of thermoresponsive materials for cell culture.

However, the utilization of PNIPAM in cell culture remains challenging, with numerous inquiries and obstacles. Presently, the fabrication of thermoresponsive substrates typically encompasses two distinct approaches: insoluble grafted films and soluble coated films. A key aspect in the preparation of insoluble grafted films lies in securely affixing PNIPAM and its derivatives onto cell culture substrates, including polystyrene and glass or porous scaffolds. This frequently necessitates the employment of sophisticated and costly techniques, like electron beam irradiation [17], plasma treatment [12], ultraviolet irradiation [14], or intricate chemical methods [6,18] for inducing the grafting of thermoresponsive components to the substrates. Unlike surface polymerization, soluble coated films can be completed through the preparation of PNIPAM polymers and the coating of polymer solutions [11,13,19,20]. The advantage is the simplicity of the film coating process, allowing for the incorporation of other functional monomers during the polymer preparation. However, due to the absence of robust linkages between molecular chains, as well as between the chains and the substrate, these coatings are susceptible to detachment or dissolution when temperatures drop below the LCST [21,22,23]. Both techniques have their shortcomings, from dependence on pricey equipment and intricate preparation procedures to the use of complex raw materials with unclear toxicity, or the insufficient stability and reversibility of the product, all of which hinder the widespread adoption and application of PNIPAM-containing materials in cell culture [24,25,26]. Thus, the potential to fabricate PNIPAM-based substrates that are thermoresponsive, controllable, stable, and reusable using simple equipment, standardized procedures, and biologically compatible synthetic materials merits further exploration.

Given the limitations of conventional PNIPAM-based substrates in terms of stability, reusability, as well as economic efficiency, this study aimed to develop a robust, reusable, and economical engineering PNIPAM-based platform for non-invasive and gentle cell detachment, particularly targeting bone marrow mesenchymal stem cells (BMMSCs), through a two-step film-forming method involving coating and grafting onto glass slides or silicon wafers. Through a comprehensive analysis of films’ surface wettability, topography, and thickness by means of contact angle analysis [27,28,29], atomic force microscopy (AFM) investigation [30,31,32], and ellipsometric measurements [33,34], the study provided valuable insights into the relationship between copolymer solution concentration and film properties. By systematically investigating PNIPAM copolymer films derived from various solution concentrations, the study sought to identify the optimal formulation that enabled good BMMSC growth at 37 °C and efficient cell detachment upon temperature reduction. Additionally, the study assessed the long-term stability and reusability of the selected PNIPAM copolymer films after two rounds of BMMSC adhesion and desorption by meticulously examining their surface texture using AFM and analyzing the elemental composition through the X-ray photoelectron spectroscopy (XPS) technique [35,36]. This study addresses critical needs in the field of biomedical engineering by providing a stable, reusable, and efficient platform for stem cell culture and harvesting, while also advancing the understanding of surface property manipulation in smart materials tailored for biomedical applications.

## 2. Results and Discussion

### 2.1. Preparation of PNIPAM-Based Films

Spin coating is the preferred method for fabricating smooth thin films, allowing precise control over the film thickness by adjusting parameters such as rotation speed and time, solution concentration, and sample volume [7,13]. In this study, the abovementioned control factors were kept constant, while the concentration of the copolymer solution was varied to achieve the copolymer films with distinct properties. A two-step polymerization-coating technique was employed. Initially, the synthesis of P(*N*-isopropylacrylamide-*co*-3-(methacryloxy) propyltrimethoxysilane-*co*-3-hydroxypropyl methacrylate) copolymers, P(NIPAM-*co*-MPS-*co*-HPMA) copolymers, also referred to as PNIPAM-based copolymers, was completed. The copolymers boasted a molar composition ratio of 20:1:1 for NIPAM, MPS, and HPMA monomers within their structure, and they exhibited an LCST hovering around 25 °C. Subsequently, the copolymer solution with concentrations ranging from 0.2 to 10 mg·mL^−1^ was uniformly spin coated onto the surfaces of glass slides or silicon wafers at a consistent speed (3000 rpm) and duration (20 s). Afterwards, the samples were placed in a vacuum oven for the annealing process. During thermal annealing, the siloxane bonds underwent demethanolization reactions with hydroxyl groups, facilitating the grafting of the copolymers onto the hydroxyl-bearing substrates and interchain bonding [37,38]. This resulted in the preparation of temperature-sensitive substrates.

**Figure 1:**
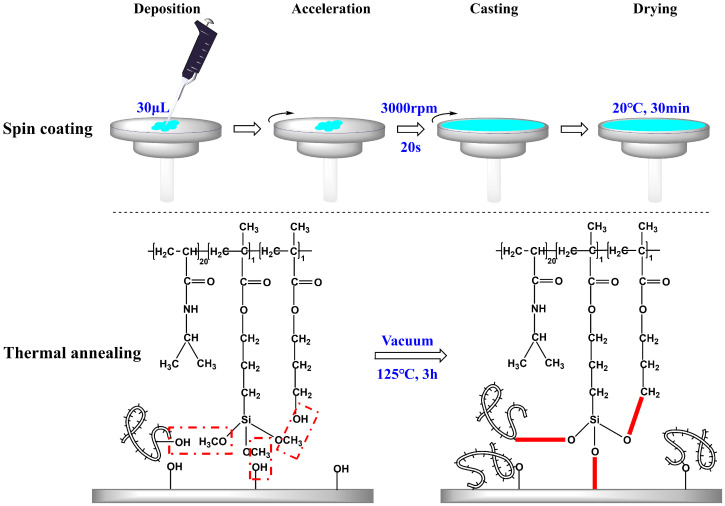
Preparation strategy of P(NIPAM-*co*-MPS-*co*-HPMA) copolymer films on glass slides or silicon wafers.

As illustrated in Figure 1, three types of crosslinking occurred simultaneously during the heating and annealing of P(NIPAM-*co*-MPS-*co*-HPMA) copolymer coating layer: bonding between copolymer siloxane bonds and substrate hydroxyl groups, intrachain bonding between siloxane bonds and hydroxyl groups, and interchain bonding between siloxane bonds and hydroxyl groups from other copolymer chains. Under the synergistic effect of these three reactions, the copolymer layer transformed from a loosely attached state to a tightly crosslinked and stable state. The covalent bonds between the copolymer film and the substrate, along with the formation of a macromolecular network structure within the film, ensured that the copolymer film remained securely adhered and resistant to dissolution, even after prolonged immersion in liquids. These attributes were crucial for cell adhesion, proliferation, and detachment.

### 2.2. Characterization of PNIPAM-Based Films

#### 2.2.1. Surface Wettability

**Figure 2:**
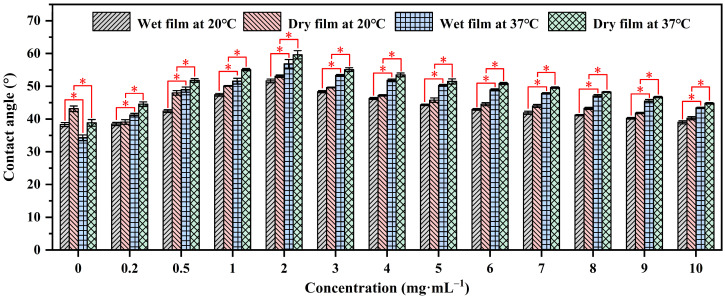
Water contact angles on P(NIPAM-*co*-MPS-*co*-HPMA) copolymer films prepared at different concentrations. * *p* < 0.05 is considered to be statistically significant.

Contact angle analysis is a technique used to evaluate the wettability of a solid surface by a liquid. It measures the angle formed between the liquid–solid interface and the tangent to the liquid surface at the point of contact, providing insights into surface properties and interactions [39,40]. The contact angles of water on P(NIPAM-*co*-MPS-*co*-HPMA) copolymer films prepared from the copolymer solutions with the concentrations ranging from 0.2 to 10 mg·mL^−1^ were measured using sessile drop method under both dry and wet conditions at 20 °C (the temperature for cell harvesting, below the LCST of the copolymers (≈25 °C)) and 37 °C (the temperature for cell growth, above the LCST), as shown in Figure 2. It was evident that regardless of the film’s state, the water contact angles of the copolymer films at 37 °C were notably higher than those at 20 °C, while the contact angles of glass slides at 37 °C were slightly lower than those at 20 °C due to the decrease in surface tension of water with increasing temperature [41]. This fully confirmed the temperature-sensitive properties of the prepared copolymer films. Additionally, the contact angles of water on dry films exceeded those on wet films under identical temperatures, as water infiltration into the film altered the surface and internal structure of the copolymer films [42,43], exhibiting the trend of *θ*_dry,37°C_ > *θ*_wet,37°C_ > *θ*_dry,20°C_ > *θ*_wet,20°C_ for a given copolymer concentration. Under dry or wet conditions at a specific temperature, the contact angles of water on the copolymer films initially increased and then decreased as the solution concentration increased, peaking at 2 mg·mL^−1^. The initial increase in contact angles was attributed to the gradual coverage of the substrate surface by P(NIPAM-*co*-MPS-*co*-HPMA) copolymers, resulting in a transition of surface properties from glass to copolymer film. However, as the concentration reached its critical value, the contact angles began to show a steady downward trajectory due to the increasing number of hydrophilic groups like amide bonds and hydroxyl groups on the surfaces of the copolymer films, fostering the accumulation of more hydrated molecules at 20 °C or 37 °C, ultimately leading to a more hydrophilic surface.

#### 2.2.2. Surface Topography

**Figure 3:**
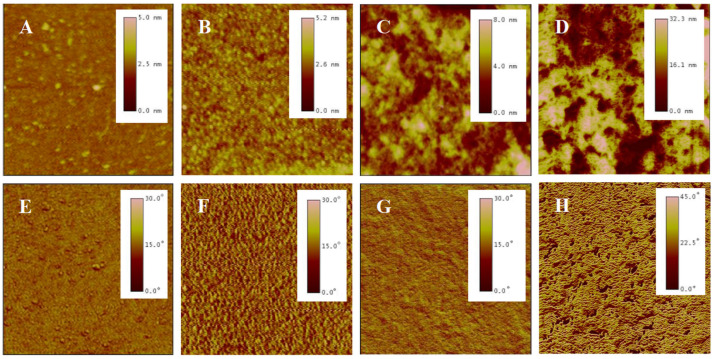
AFM topography (top) and phase (bottom) images of P(NIPAM-*co*-MPS-*co*-HPMA) copolymer films prepared at the concentrations of 0.2 mg·mL^−1^ ((**A**,**E**), 2 µm scan), 1 mg·mL^−1^ ((**B**,**F**), 2 µm scan), 5 mg·mL^−1^ ((**C**,**G**), 2 µm scan), and 10 mg·mL^−1^ ((**D**,**H**), 10 µm scan).

AFM is a high-resolution imaging tool that explores surfaces at nanoscale. It employs a probe tip to scan the surface, detecting atomic-scale interactions and rendering three-dimensional topographic maps, revealing surface details unattainable by optical microscopes [44]. A meticulous analysis was conducted on the surface morphology of P(NIPAM-*co*-MPS-*co*-HPMA) copolymer films via AFM. Figure 3 presents the topography and phase images of P(NIPAM-*co*-MPS-*co*-HPMA) copolymer films formed under different concentrations. Overall, alterations in concentrations manifested themselves through distinct surface features of the copolymer films, particularly in terms of the number, height, and distribution of peaks. Figure 3A,E exhibit the copolymer film produced from 0.2 mg·mL^−1^ solution, displaying a remarkably smooth surface (roughness *R*_q_ ≈ 0.2 nm) adorned with a sparse scattering of tiny round protrusions (peak height *H*_p_ = 2.5~5 nm), indicating that only a small amount of the copolymer particles deposited on the substrate under a relatively dilute concentration. As indicated by dynamic light scattering analyses, the average hydrodynamic diameter of P(NIPAM-*co*-MPS-*co*-HPMA) copolymer single chains measured about 10 nm [45], whereas the observed peak heights were merely 2.5~5 nm, suggesting the successful grafting of copolymer single chains onto the substrate via the demethanolation reaction between siloxane bonds and multiple hydroxyl sites [37,38]. As the solution concentration rose to 1 mg·mL^−1^, as depicted in Figure 3B,F, a more evenly spaced distribution of round protrusions emerged on the substrate surface, maintaining similar peak heights compared to the 0.2 mg·mL^−1^ sample (*R*_q_ ≈ 0.3 nm, *H*_p_ = 2.5~5 nm). This implied that the copolymers were progressively covering the surface and initiating the formation of interconnections at a uniform level. As seen in Figure 3C,G, the copolymer film prepared from 5 mg·mL^−1^ solution displays an uneven surface (*R*_q_ ≈ 1.4 nm), with localized copolymer aggregations featuring peak heights of 4~8 nm. This phenomenon arose from the formation of interconnected multi-layers of copolymer molecular chains at higher concentrations, while some regions revealed the exposed substrate surface. The copolymer film derived from 10 mg·mL^−1^ solution (Figure 3D,H) displayed an even rougher and unevenly distributed surface (*R*_q_ ≈ 7.0 nm, *H*_p_ = 16~32 nm), resembling a granular distribution pattern of “mountains and valleys”. In essence, when the copolymer solution concentration was low, P(NIPAM-*co*-MPS-*co*-HPMA) copolymers began to deposit on and gradually cover the substrate. As the concentration further increased, the copolymer layers accumulated, resulting in thicker and rougher copolymer films, which aligned well with the experimental results of surface contact angles.

#### 2.2.3. Film Thickness

**Figure 4:**
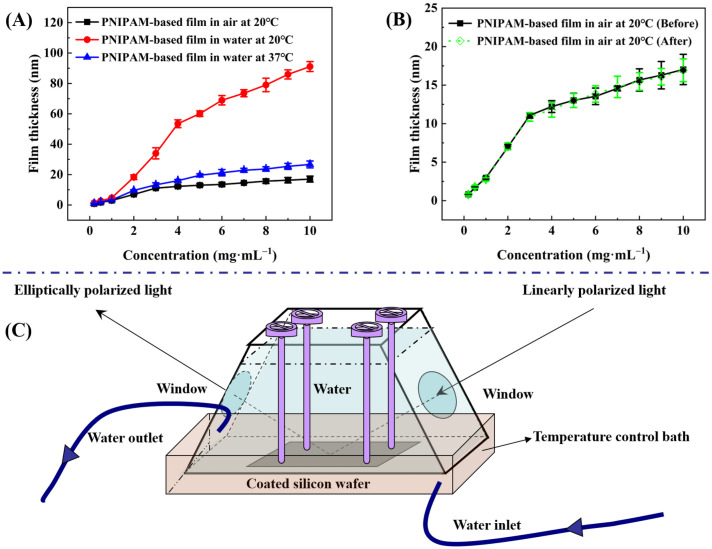
Thickness of P(NIPAM-*co*-MPS-*co*-HPMA) copolymer films prepared at different concentrations under various treatments (**A**), comparison of film thickness before and after treatment (**B**), and a unique measurement chamber with water storage and temperature regulation capabilities (**C**).

Ellipsometry measures the change in polarization state of light reflected from a sample surface. By analyzing this change, the thickness of thin films can be accurately determined [46,47]. With the aid of an ellipsometer bearing a specialized chamber that incorporates both water storage and temperature control functionalities (see Figure 4C), measurements were taken to determine the thicknesses at the same site of P(NIPAM-*co*-MPS-*co*-HPMA) copolymer films that originated from different copolymer concentrations, both in air at 20 °C and in ultrapure water at 20 °C and 37 °C. Prior to each coating session, the oxide layer thicknesses of thoroughly cleansed silicon wafers were precisely measured to guarantee accuracy, and these values were held constant for the subsequent analysis of copolymer film thickness. Data-fitting techniques allowed for an estimation of the refractive index of P(NIPAM-*co*-MPS-*co*-HPMA) copolymer film at 1.470 under 20 ℃ and 632.8 nm wavelength, while the refractive indices stood at 3.875 for silicon (20 ℃, 632.8 nm) and 1.459 for silica (20 ℃, 632.8 nm), respectively. The difference in refractive indices between various materials facilitated the division of different layers.

Firstly, the thicknesses of P(NIPAM-*co*-MPS-*co*-HPMA) copolymer dry films across the solution concentration range spanning from 0.2 to 10 mg·mL^−1^ established a correlation between copolymer concentration and corresponding film thickness (ranging from 0.8 to 17.0 nm). Figure 4A reveals that, within a solution concentration range of 0.2 to 3 mg·mL^−1^, the film thickness increased rapidly with copolymer concentration, whereas from 3 to 10 mg·mL^−1^, the growth rate tapered off. This suggests that the copolymers gradually and completely covered the substrate surface, simultaneously undergoing crosslinking from a single layer to multiple layers of molecular chains. Before thermal annealing, higher solution concentration led to a thicker deposition of copolymers on silicon wafers. However, after annealing, due to steric hindrance, covalent crosslinking became increasingly difficult within and between molecules, resulting in a slower growth trend of film thickness at elevated copolymer concentrations.

Figure 4A concurrently illustrates the alteration in the thickness of P(NIPAM-*co*-MPS-*co*-HPMA) copolymer films upon immersion in ultrapure water at both 20 °C and 37 °C. In essence, regardless of the temperature, the trend in thickness variation observed in the copolymer films in water mimicked the pattern seen in dry copolymer films with varying concentrations. Notably, below a critical concentration of 3 mg·mL^−1^, there was a relatively steep increase in film thickness, which tapered off above this concentration. However, the extent of thickness variation was markedly influenced by temperature. Taking P(NIPAM-*co*-MPS-*co*-HPMA) copolymer films prepared at the concentration of 10 mg·mL^−1^ as an example, the film thickness in air at 20 °C was approximately 17.0 nm. Upon immersion in ultrapure water of 20 °C, the film thickness significantly swelled to 91.1 nm, yielding a swelling ratio of 5.36 times. Conversely, the film thickness contracted to around 26.7 nm at 37 °C, exhibiting a reduced swelling ratio of 1.57 times. These findings underscored the thermoresponsivity of P(NIPAM-*co*-MPS-*co*-HPMA) copolymer films. Upon immersion in water at 20 °C, water molecules promptly infiltrated the porous structure of PNIPAM-based films, forming hydrated hydrogen bonds with hydrophilic moieties, leading to pronounced swelling [48,49]. However, at 37 °C, hydrophobic interactions among isopropyl groups dominated, and hydrated hydrogen bonds partially transformed into non-aqueous bonds, ultimately maintaining the collapsed state of the copolymer films [48,49]. Upon vacuum drying after immersion and temperature variations, the film thickness in air at 20 °C returned to approximately 16.9 nm (see Figure 4B), comparable to the original dry film, demonstrating the stability of P(NIPAM-*co*-MPS-*co*-HPMA) copolymer films.

Although the thickness changes in the copolymer films formed from 1 mg·mL^−1^ in aqueous solutions were less pronounced compared to those prepared at 10 mg·mL^−1^ solution, the fundamental trends remained similar. Starting with a dry film thickness of around 3.0 nm in air at 20 °C, the film thickness increased to approximately 4.7 nm upon immersion in ultrapure water at 20 °C, exhibiting a swelling ratio of about 1.57 times. When the temperature was elevated to 37 °C, the film thickness decreased to nearly 3.3 nm, resulting in a diminished swelling ratio of 1.10 times. Even considering the original dry film thickness, the observed degrees of swelling and shrinking were remarkable. As evidenced by the experimental outcomes presented in Section 2.2.4, even the swelling of P(NIPAM-*co*-MPS-*co*-HPMA) copolymer films derived from 1 mg·mL^−1^ solution was sufficient to facilitate the detachment of adhered BMMSCs.

The crosslinking degree in P(NIPAM-*co*-MPS-*co*-HPMA) copolymer chains strongly correlated with the concentration used during preparation. In dilute solutions at lower concentrations, crosslinking predominantly occurred among isolated chains, with weaker intermolecular interactions that limited extensive crosslinking. For instance, the copolymer films prepared using a solution concentration of 0.2 mg·mL^−1^, upon thermal annealing, displayed the localized and crosslinked clusters where individual chains were deposited, visible as scattered dots in Figure 3A. In contrast, at higher concentrations (e.g., 5 mg·mL^−1^), numerous copolymer chains stacked densely, enabling not only adhesion to the substrate but also interchain coupling during annealing, resulting in a robust polymer network. This crosslinking degree crucially influenced the swelling behavior of the copolymer films. Typically, a higher crosslinking degree led to lower swelling [50,51]. Table 1 summarizes the swelling ratio of P(NIPAM-*co*-MPS-*co*-HPMA) copolymer films prepared from different concentrations at 20 °C and 37 °C in ultrapure water. Below or at 1 mg·mL^−1^, the copolymer chains were unable to fully form a cohesive network film, resulting in a low swelling ratio at both 20 °C and 37 °C. However, the copolymer films prepared with 0.2 mg·mL^−1^ solution, with the lowest crosslinking in this range, displayed the highest swelling ratio. According to Figure 3B, the copolymer films formed from 1 mg·mL^−1^ solution exhibited nascent features of a monomolecular layer. As the preparation concentration rose up to 4 mg·mL^−1^, the films evolved from a tightly packed single layer to a multi-layer structure with a looser arrangement, leading to reduced crosslinking and enhanced swelling ratio at both temperatures. When the concentration increased to 5 mg·mL^−1^ or higher, the swelling ratio slightly increased at 20 °C, and it stabilized at 1.50+ under 37 °C. This arose from increased spatial constraints among copolymer chains at higher concentrations, limiting significant thickness growth (see Figure 4) and achieving relative balances in water binding (swelling) or water expulsion (shrinking) capabilities.

#### 2.2.4. Screening of PNIPAM-Based Films Suitable for BMMSCs

**Figure 5:**
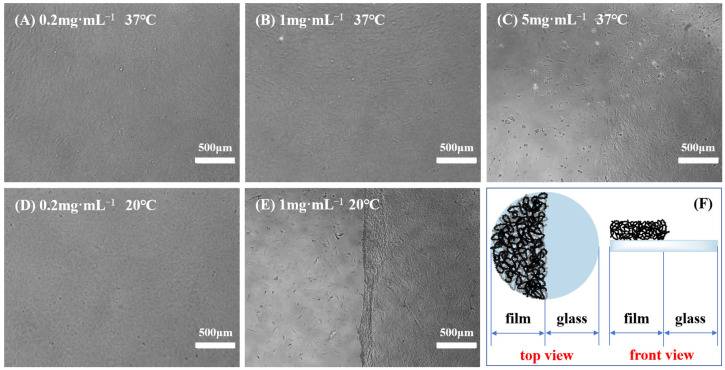
Typical cases of BMMSC adhesion and detachment on the thermoresponsive half-film slides prepared from 0.2 mg·mL^−1^ solution (**A**,**D**), 1 mg·mL^−1^ solution (**B**,**E**) and 5 mg·mL^−1^ solution (**C**) before and after cooling treatment, and schematic of thermoresponsive half-film slide (**F**).

Previous studies showed that not all PNIPAM-based substrates were suitable for cell culture and harvest, indicating that adherent cells exhibited distinct preferences towards temperature-sensitive substrates [10,11]. Variables, such as the grafting thickness (grafting ratio) of polymers and the ratio of PNIPAM and other functional materials, can alter the surface wettability and microstructure of culture substrates, thereby affecting cell adhesion and subsequent physiological activities. Moreover, diverse cell types possess varying demands for the properties of polymer films, and even identical cell types may have different requirements for film characteristics [52,53]. Consequently, after the creation of a novel PNIPAM-based substrate, it is imperative to initially determine the appropriate range of film properties conducive to cell adhesion and detachment. To identify a temperature-responsive culture substrate tailored for BMMSC adhesion and detachment, the impact of surface wettability, surface roughness, and film thickness of P(NIPAM-*co*-MPS-*co*-HPMA) copolymer films on BMMSC adhesion and detachment was investigated in this study. Specifically, BMMSCs were inoculated onto a series of temperature-responsive half-film slides, crafted from varying solution concentrations (one side comprised temperature-responsive copolymer film, while the other side consisted of glass, as depicted in Figure 5F). Subsequently, cell growth on these slides was monitored, encompassing adhesion, spreading, migration, and proliferation. Once the cells reached confluence, their detachment during temperature reduction was observed in real time.

Figure 5 displays several typical cases of BMMSC adhesion and detachment on the temperature-sensitive half-film slides before and after cooling treatment. When using the half-film slides prepared with 0.2 mg·mL^−1^ solution, there was no significant difference in the adhesion behavior of BMMSCs on both thermoresponsive film side and glass side. Regardless of the temperature being 37 °C or 20 °C, BMMSCs displayed typical spindle-shaped morphology. Nevertheless, upon the temperature falling below the LCST, the cells failed to detach from the film side, revealing that the film thickness and surface wettability were only conducive to cell adhesion, not detachment. On the copolymer half-film slides formulated from 1 mg·mL^−1^ solution, the adhesion and growth levels of BMMSCs on both sides were comparable and the cells showed good growth patterns at 37 °C. However, upon cooling to below the LCST and replacing with cold fresh culture medium, BMMSCs on the film side detached within minutes, while the cells on the glass side remained firmly attached to the culture substrate, leaving a distinct boundary. This demonstrated that the copolymer films could be used for BMMSC attachment and detachment, as the overall thickness and surface wettability at 37 °C were suitable for cell adhesion and growth, while the changes in thickness and wettability at 20 °C were sufficient to trigger cell detachment. In the 5 mg·mL^−1^ group, BMMSCs grew and proliferated well on the glass side but adhered scarcely and did not spread on the film side. A clear boundary was also observed at 37 °C, indicating that the copolymer films were unfavorable for cell growth and even more so for detachment.

When utilizing a thinner grafting layer or reduced grafting quantity, the alterations in surface wettability and volume phase transition of PNIPAM-based films with temperature are inadequate to remove adherent cells. Conversely, as the grafting thickness or amount increased to a specific threshold, the surface becomes suitable for cell adhesion at 37 °C, and the extent of change in film properties after cooling treatment enables cell detachment, making it an optimal choice for cell harvesting. Further increasing the grafting thickness or grafting amount leads to a surface that no longer favors cell adhesion, resulting in the cell suspending in culture medium due to adhesion site loss, ultimately culminating in cell apoptosis or even death. Based on the overall experimental results, BMMSCs were able to adhere to and proliferate on P(NIPAM-*co*-MPS-*co*-HPMA) copolymer films prepared from 0.5 to 2 mg·mL^−1^ solutions (featuring dry film contact angles spanning from 51.8° to 59.6° and dry film thicknesses ranging from 1.7 to 7.0 nm), and they could successfully detach from these copolymer films. Considering the necessity for a temperature-responsive culture substrate to foster cell adhesion and shorten cell detachment time simultaneously [54], this study identified P(NIPAM-*co*-MPS-*co*-HPMA) copolymer films prepared from 1 mg·mL^−1^ solution as the appropriate culturing substrates for BMMSCs. The substrates exhibited a static water contact angle of approximately 55.1° and a film thickness of about 3.0 nm in its dry state.

### 2.3. Reusability Evaluation of PNIPAM-Based Films

#### 2.3.1. Stability of Film Properties

Given that this study deemed P(NIPAM-*co*-MPS-*co*-HPMA) copolymer films prepared at 1 mg·mL^−1^ solution as the optimal culture substrates for BMMSCs, an examination was conducted on the stability of the copolymer films’ properties at this specific concentration. The newly formulated copolymer films were individually stored in sealed, dry, and sterile environments at the temperatures of 4 °C, 20 °C, and 37 °C for a duration of four weeks, respectively. Following this period, the copolymer films were retrieved for evaluation of their surface wettability and film thickness. The findings indicated that the fluctuations in the two performance parameters remained approximately 1% (see Table S1), conclusively demonstrating the storage stability of P(NIPAM-*co*-MPS-*co*-HPMA) copolymer films. Additionally, the copolymer films were immersed in ultrapure water and underwent three rounds of alternating heating and cooling procedures. Subsequently, the copolymer films were vacuum-dried and reassessed for surface wettability and film thickness. Notably, the deviations in the two indicators remained within 3% (see Table S1), thereby proving the operational stability and reusability of P(NIPAM-*co*-MPS-*co*-HPMA) copolymer films.

#### 2.3.2. Surface Topography of PNIPAM-Based Films after Cell Culture and Harvest

Despite the employment of the spin coating method in this study for fabricating temperature-responsive copolymer films, the copolymer–ethanol solution can be adsorbed and fixed onto various shapes and sizes of culture substrates containing hydroxyl groups, such as microcarriers or hollow fibers, through techniques such as rinsing, spraying, dipping, and thermal annealing. Given the complicated procedures for the assembly and sterilization of the corresponding bioreactors, the reusability of these temperature-sensitive materials was of particular importance. Therefore, examining the reusability of the thermosensitive planar films facilitated the promotion and application of P(NIPAM-*co*-MPS-*co*-HPMA) copolymers in three-dimensional culture systems. In this study, BMMSCs were cultured and harvested on P(NIPAM-*co*-MPS-*co*-HPMA) copolymer films prepared from 1 mg·mL^−1^ solution, undergoing two rounds of culture and harvest processes. Following each harvesting session, the cells were capable of achieving confluence on the same copolymer films at 37 °C, and most of the cells detached spontaneously from the films when the temperature was reduced to 20 °C. This revealed no significant difference in the growth and detachment of cells on the same PNIPAM-based films after different cultivation cycles, highlighting the structural stability and functional durability of the copolymer films [55,56]. It also demonstrated that P(NIPAM-*co*-MPS-*co*-HPMA) copolymer films could be repeatedly used for culture and harvest of adherent cells.

**Figure 6:**
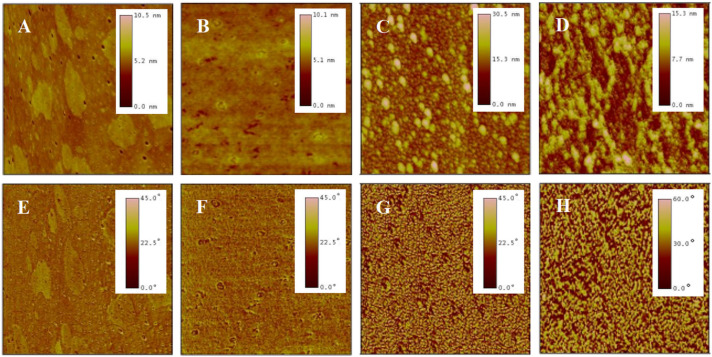
AFM topography (top) and phase (bottom) images of bare glass slide (**A**,**E**), P(NIPAM-*co*-MPS-*co*-HPMA) copolymer films (1 mg·mL^−1^) (**B**,**F**), and copolymer films (1 mg·mL^−1^) treated by one (**C**,**G**) and two (**D**,**H**) rounds of BMMSC attachment and detachment. The scan size is 2 µm for all images.

Before cell seeding, protein components in the culture medium, such as fibronectin, laminin, collagen, and albumin, adsorb onto the surfaces of the temperature-sensitive copolymer films, promoting rapid cell localization and adhesion. As the cells adhere and grow, they gradually form their own extracellular matrix network around them through autocrine effects. After cell detachment by reducing temperature, a certain amount and variety of extracellular matrix proteins remain on the surfaces of PNIPAM-based films [12,57,58,59], leading to the changes in surface morphology. This study examined the morphological changes in P(NIPAM-*co*-MPS-*co*-HPMA) copolymer films after several cycles of BMMSC cultivation by means of AFM. Figure 6 shows AFM images of bare glass slide (Figure 6A,E), P(NIPAM-*co*-MPS-*co*-HPMA) copolymer film prepared at 1 mg·mL^−1^ solution (Figure 6B,F), and the copolymer film fabricated from the same solution concentration after one (Figure 6C,G) and two (Figure 6D,H) rounds of BMMSC culture and harvest. The image revealed a relatively rough surface on bare glass slide, exhibiting fine pores and scattered spots (*R*_q_ ≈ 0.52 nm, *H*_p_ ≈ 10.5 nm). In contrast, the copolymer film formed from 1 mg·mL^−1^ solution was comparatively smoother (*R*_q_ ≈ 0.46 nm, *H*_p_ ≈ 10.1 nm), attributed to the copolymer particles filling the pores in the glass slide and obscuring its surface imperfections. There were notable variations in the surface morphology of the temperature-responsive films after cell attachment and detachment compared to bare copolymer films. The copolymer film after one round of BMMSC detachment exhibited an uneven peak distribution and a rougher surface (*R*_q_ ≈ 3.3 nm, *H*_p_ ≈ 30 nm). However, following two consecutive rounds of BMMSC detachment, the film exhibited an increased peak density, with a substantial reduction in the highest peak and overall roughness (*R*_q_ ≈ 2.4 nm, *H*_p_ ≈ 15 nm). This suggested that cells densely clustered in specific areas of the copolymer films, leaving behind more protein residues in the first cycle. In the second cycle, BMMSCs carried away some residual proteins while depositing additional proteins in other film areas. It was speculated that with increasing cell culture rounds, an integrated and uniform protein layer would gradually form on the surface of the copolymer film. These findings indicated that the continuous cell culture and harvest process altered the surface morphology of P(NIPAM-*co*-MPS-*co*-HPMA) copolymer films due to protein deposition but did not substantially impair the temperature-responsive properties of the copolymer films. Moreover, these residual proteins facilitated rapid cell localization and effective adhesion during subsequent cell inoculation, indirectly demonstrating the reusability of the copolymer films.

#### 2.3.3. Elemental Composition of PNIPAM-Based Films after Cell Culture and Harvest

XPS is an analytical tool with exceptional surface sensitivity, enabling precise identification and characterization of elements residing on material surfaces. It functions by irradiating a specimen with X-rays, stimulating electrons to vacate their atomic orbitals, subsequently measuring the kinetic energy of these emitted photoelectrons. This process reveals valuable information about the elemental compositions, chemical states, and bonding configurations of the superficial layers of the sample, typically within the top 10 nm [60,61].

**Figure 7:**
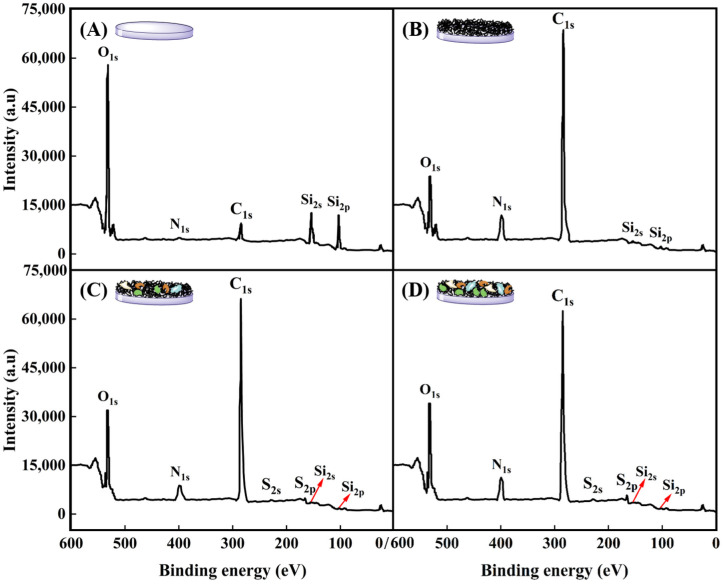
XPS spectra of bare glass slide (**A**), P(NIPAM-*co*-MPS-*co*-HPMA) copolymer films (1 mg·mL^−1^) (**B**), and copolymer films (1 mg·mL^−1^) treated by one (**C**) and two (**D**) rounds of BMMSC attachment and detachment.

Surface compositions of glass slide, P(NIPAM-*co*-MPS-*co*-HPMA) copolymer films (1 mg·mL^−1^), and copolymer films (1 mg·mL^−1^) treated upon several cell attachments and detachments were determined by XPS analysis, as displayed in Figure 7 and summarized in Table 2. For bare glass slide, the C_1s_ (284.7 eV), O_1s_ (531.5 eV), N_1s_ (399.8 eV), Si_2s_ (153.2 eV), and Si_2p_ (102.4 eV) core levels were detected in Figure 7A, which were in agreement with previous studies [62,63]. Upon grafting P(NIPAM-*co*-MPS-*co*-HPMA) copolymers onto the glass slide surfaces, notable increases in the relative atomic percentages of C from 9.2% to 70.6% and N from 1.3% to 10.4% were observed, while O and Si levels decreased significantly from 65.1% to 17.3% and from 24.4% to 1.7%, respectively, as shown in Figure 7B. The measured copolymer film composition slightly deviated from the predicted values based on the comonomer stoichiometry of NIPAM-*co*-MPS-*co*-HPMA, which theoretically comprised 73.7% C, 15.0% O, 10.8% N, and 0.5% Si (excluding H). As visualized in Figure 6B,F, incomplete coverage of the glass surface by the copolymers, leaving some uncovered areas, indicates potential influence from the glass elemental composition on the results. Nevertheless, the pronounced elevation in C and N levels alongside the marked decline in O and Si conclusively demonstrated the successful grafting of PNIPAM-based copolymers onto the glass surface.

Figure 7C,D reveal the emergence of two new and weak elemental peaks attributed to S_2s_ (228.0 eV) and S_2p_ (164.5 eV) on P(NIPAM-*co*-MPS-*co*-HPMA) copolymer films (1 mg·mL^−1^) after one or two cycles of BMMSC adhesion and detachment and absence on both bare glass slides and copolymer films. The observed S peaks were likely attributed to the amino groups derived from proteins on the copolymer films used by BMMSCs. Notably, the C_1s_ and N_1s_ peaks on the used copolymer films were less intense compared to those on bare PNIPAM-based films, yet exhibited a markedly elevated O_1s_ peak. Following BMMSC culture and subsequent removal via cooling treatment, the surfaces were generally found to be enriched in O, with reduced levels of C, N, and Si. These findings suggested the presence of ECM protein biomolecules, either secreted by BMMSCs or originating from culture medium, adhering to the surfaces after cell culture and harvesting [12,58]. Table 1 further highlights that the atomic percentages of O, N, and S in the copolymer films subjected to two rounds of cell attachment and detachment are slightly higher than those undergoing a single round, whereas the trend is reversed for C percentage. This fully demonstrated an increase in proteins adhering to P(NIPAM-*co*-MPS-*co*-HPMA) copolymer films with each successive cycle of cell culture and harvesting.

## 3. Materials and Methods

### 3.1. Materials

P(NIPAM-*co*-MPS-*co*-HPMA) copolymers were synthesized through free radical polymerization using 2,2-azobisisobutyronitrile (AIBN) as an initiator, according to our prior investigations [49], featuring the LCST of approximately 25 °C as measured by dynamic light scattering (Nano ZS, Malvern Instruments Ltd., Malvern, UK), and the molar composition ratio of 20:1:1 among the monomers NIPAM, MPS, and HPMA as identified by nuclear magnetic resonance hydrogen spectrum (^1^H-NMR, Bruker AV 400 NMR, Bruker Corporation, Fällanden, Switzerland). The silicon wafers (100) from Compart Technology Ltd. (Tamworth, UK) and the glass slides (Φ 13) from VWR International, LLC (West Chester, PA, USA) served as the substrates after being thoroughly cleaned with detergent followed by ultrapure water with a conductivity of 0.055 μS·cm^−1^ and a resistivity of 18.2 MΩ·cm at 25 °C. Low-glucose Dulbecco’s modified Eagle’s medium (LG-DMEM) with 1000 mg/L glucose and L-glutamine, fetal bovine serum (FBS, USA origin), and anhydrous ethanol (≥99.5%, anhydrous, 200 proof) were obtained from Sigma-Aldrich (St. Louis, MO, USA). Rat-derived BMMSCs were supplied by ATCC (Manassas, VA, USA).

### 3.2. Film Preparation

The concentration of P(NIPAM-*co*-MPS-*co*-HPMA) copolymers in anhydrous ethanol was adjusted to between 0.2 and 10 mg·mL^−1^. This mixture was agitated thoroughly for 12 h at 20 °C. Following this, 0.2 μm pore-sized filters were utilized to remove any insoluble particles from the copolymer solution. Silicon wafers or glass slides were securely placed on the sample holder of a Laurell spin coater (WS-400BZ-6NPP/LITE, Laurell Technologies Corp., North Wales, PA, USA), and 30 µL of the copolymer solution was carefully dispensed onto the center of each substrate. With a rotational speed set at 3000 rpm for 20 s, the copolymers were evenly spread over the surface. The coated layers were allowed to stand at 20 °C for at least 30 min, then transferred to a vacuum oven, and annealed at 125 °C for 3 h to encourage the bonding of siloxane groups with hydroxyl groups and facilitate the evaporation of residual anhydrous ethanol. Afterwards, the samples were immersed in anhydrous ethanol and ultrapure water separately, for 60 min each, to purge any unreacted monomers or unconnected copolymers. Finally, the samples underwent thorough rinsing with ultrapure water and were vacuum-dried at 20 °C for further use.

### 3.3. Film Performance

#### 3.3.1. Surface Contact Angle Analysis

The contact angles of dry and wet films of P(NIPAM-*co*-MPS-*co*-HPMA) copolymer were evaluated at 20 °C and 37 °C through sessile drop approach utilizing a KRüSS DSA100 Drop Shape Analyzer (Hamburg, Germany). Prior to testing, the dry films underwent vacuum drying at 20 °C for 12 h; whereas, the wet films were immersed in ultrapure water for 10 min and subsequently dried using filter paper to remove excess water. To ensure precise temperature control, a temperature regulator was employed, with the temperature fluctuation of ±0.5 °C. The surface contact angles were determined using Drop Shape Analysis Software (V1.9-03). Each experiment was replicated three times, and the results were presented in the form of mean values, accompanied by standard deviations.

#### 3.3.2. Surface Morphology Analysis

The surface morphology of P(NIPAM-*co*-MPS-*co*-HPMA) copolymer films, prepared at varying copolymer concentrations, was scrutinized utilizing the tapping mode of an AFM (Nanoscope IIIa, Digital Instruments, Santa Barbara, CA, USA). The employed nitride silicon probe possessed a resonant frequency below 270 kHz, with a tip radius of around 20 nm and a scanning rate at 2.959 Hz. For each specimen, three random locations were chosen, and the scanning was performed across various scales, spanning from 500 nm to 10 µm. Surface characteristics, including roughness and height, were examined through Nanoscope III software (V5.31r1), with the experimental outcomes expressed as the average values.

#### 3.3.3. Film Thickness Analysis

To ensure the accuracy of measurement, prior to film fabrication, the oxide layer thicknesses on the thoroughly cleaned silicon wafers were initially gauged via an ellipsometer (UVISEL, Horiba Jobin-Yvon, Longjumeau Cedex, France), following the measurement procedure described in the cited literature [64]. Subsequently, the thicknesses of P(NIPAM-*co*-MPS-*co*-HPMA) copolymer films derived from different copolymer concentrations were assessed using the ellipsometer in both air at 20 °C and ultrapure water at varying temperatures (20 °C and 37 °C).

To quantify the thickness variations in P(NIPAM-*co*-MPS-*co*-HPMA) copolymer films at the same location in air and ultrapure water, a unique measurement chamber incorporating water storage and temperature regulation capabilities was developed and is depicted in Figure 4C. The detailed measurement protocol was as follows: Initially, the silicon wafer with copolymer film was firmly attached inside the measurement chamber, which was subsequently placed on the designated testing platform. The angle of the copolymer film was adjusted to achieve perfect horizontality, whereupon its thickness in air at 20 °C was measured. Subsequently, ultrapure water maintained at 20 °C was delicately introduced into the chamber using a syringe, and the system was allowed to equilibrate for 10 min prior to assessing the film thickness in water. Following this, the heating device was activated to elevate the water temperature within the chamber to 37 °C. After another stabilization period of 10 min, the film thickness in ultrapure water of 37 °C was recorded. Upon completion of the testing, the copolymer film was removed and dried in a vacuum oven at 20 °C. Finally, the film thickness after water immersion and temperature exposure was reassessed. The experimental data were analyzed and processed using DeltaPsi II software (V2.7). Three random samples were selected for each set of tests, and the mean value ± standard deviation was considered as the representative experimental outcome.

#### 3.3.4. Film Screening for BMMSCs

To thoroughly evaluate the variations in cellular attachment and detachment between P(NIPAM-*co*-MPS-*co*-HPMA) copolymer films and glass slides, and to determine the optimal thermosensitive substrates for the cultivation and recovery of BMMSCs, the thermosensitive half-film slides with half being glass and half being copolymer film were designed and prepared, as illustrated in Figure 5F. A series of half-film slides with different surface wettabilities, morphologies, and thicknesses were created by distinct copolymer concentrations ranging from 0.2 to 10 mg·mL^−1^. These half-film slides were sterilized using ultraviolet (UV) irradiation and then transferred into 24-well cell culture plates for further use. BMMSCs were seeded onto the culture plates with different thermosensitive half-film slides at a cell density of 5 × 10^4^ cells·mL^−1^. The cells were cultured under 37 °C and 5% CO_2_ until they achieved confluence. Then, the culture plates were shifted to a sterile setting at 20 °C, and the initial culture medium was discarded. Fresh and cold LG-DMEM medium (lacking FBS, at approximately 20 °C) was introduced, and the cells were carefully removed using a pipette for a duration of about 5 min. The resulting cell suspensions were aspirated. The aforementioned process corresponded to cooling-induced cell detachment method. One or multiple groups of the copolymer films that exhibited satisfactory cell adherence at 37 °C and facilitated easy detachment at lower temperatures were selected. These chosen copolymer films would serve as the optimal substrates for the culture and harvest of BMMSCs.

### 3.4. Film Reusability

#### 3.4.1. Film Surface Topography upon Cell Attachment and Detachment

Based on the protocol of Section 3.3.4, BMMSCs were seeded onto the optimal P(NIPAM-*co*-MPS-*co*-HPMA) copolymer films prepared at 1 mg·mL^−1^ solution. After the cells grew to confluence, cooling-induced cell detachment method was employed to acquire and collect adherent cells. This collection process was repeated 2 to 3 times, aiming to ensure minimal or no cell adhesion on the copolymer films. Afterwards, the recovered cells were reintroduced onto the previously utilized thermosensitive films with a cell density of 5 × 10^4^ cells·mL^−1^. The entire process of cell culture and harvesting on the same film was repeated twice. AFM was also utilized to examine the surface topography of the copolymer films after several rounds of cell attachment and detachment.

#### 3.4.2. Film Surface Element upon Cell Attachment and Detachment

To investigate the elemental composition of the surfaces of P(NIPAM-*co*-MPS-*co*-HPMA) copolymer films subjected to varying treatments, XPS measurements were conducted utilizing a PHI-5000C ESCA system (PerkinElmer, Shelton, CT, USA). This analysis employed a focused monochromatic Al-Kα excitation source of 1486.6 eV. A comprehensive survey of spectra was captured at a pass energy of 140 eV, while high-resolution spectra focusing on specific elements (O_1s_, C_1s_, N_1s_, Si_2s_, Si_2p_, S_2s_, and S_2p_) were recorded at a pass energy of 25 eV. The calibration of all binding energies was automatically adjusted to align the C_1s_ photoelectron peak at 284.7 eV. The relative abundances of the primary elements were automatically computed by equipment-associated Multipak software (V9.3.0). The reported data represented an average derived from analyzing three randomly selected regions on each sample.

## 4. Conclusions

This study designed and fabricated P(NIPAM-*co*-MPS-*co*-HPMA) copolymer films with improved physicochemical properties, specifically tailored for the delicate culture needs of BMMSCs. Validation through surface wettability, surface morphology, and film thickness affirmed high thermosensitivity, excellent controllability, robust stability, and good reusability of PNIPAM-based films, which underscored their versatility and potential for widespread application in stem cell engineering and tissue engineering. The study successfully identified and optimized the copolymer films derived from 1 mg·mL^−1^ solution as the optimal substrates for BMMSC cultivation. The enhanced films not only facilitated efficient cell attachment and proliferation during culture phase but also ensured gentle and effective cell detachment upon harvesting. Additionally, examination of the copolymer film surfaces after multiple cycles of cell attachment and detachment, encompassing morphological observation and elemental analysis, suggested the presence of extracellular matrix proteins adhering to the film surfaces. The residual proteins expedited subsequent cell seeding and effective adhesion, implicitly attesting to the feasibility and reusability of the copolymer films, a characteristic that held immense implications for enhancing the scalability and economic viability of stem cell therapies. This study presents a significant step forward in the development of advanced biomaterials tailored for biomedical technologies.

## Figures and Tables

**Table 1:** Swelling ratio of P(NIPAM-*co*-MPS-*co*-HPMA) copolymer films prepared at different concentrations at 20 °C and 37 °C in ultrapure water.

**Copolymer Concentration (mg·mL^−1^)**	**0.2**	**0.5**	**1**	**2**	**3**	**4**
Swelling ratio (20 °C)	1.74	1.65	1.57	2.63	3.07	4.38
Swelling ratio (37 °C)	1.20	1.18	1.10	1.21	1.25	1.31
**Copolymer Concentration (mg·mL^−1^)**	**5**	**6**	**7**	**8**	**9**	**10**
Swelling ratio (20 °C)	4.62	5.09	5.18	5.22	5.27	5.36
Swelling ratio (37 °C)	1.50	1.53	1.55	1.51	1.56	1.57

Note: The data are expressed as the average values.

**Table 2:** Surface elemental composition of P(NIPAM-*co*-MPS-*co*-HPMA) copolymer films upon different treatments determined by XPS.

Sample	C	O	N	Si	S
Glass slide	9.2%	65.1%	1.3%	24.4%	0
Copolymer films (1 mg·mL^−1^, measured)	70.6%	17.3%	10.4%	1.7%	0
Copolymer films (1 mg·mL^−1^, calculated)	73.7%	15.0%	10.8%	0.5%	0
Copolymer films (1 mg·mL^−1^) treated by one round	68.0%	25.2%	5.9%	0.3%	0.6%
Copolymer films (1 mg·mL^−1^) treated by two rounds	64.2%	27.4%	7.2%	0.2%	1.0%

Note: The data are expressed as the average values.

## Data Availability

All data are contained within the article.

## Supplementary Materials

The following supporting information can be downloaded at: https://www.mdpi.com/article/10.3390/molecules29184481/s1, Table S1: Performance stability of P(NIPAM-*co*-MPS-*co*-HPMA) copolymer films (1 mg·mL^−1^) after different treatment.

## References

[1] Luo Y., Gao Y. (2024). Potential role of hydrogels in stem cell culture and hepatocyte differentiation. Nano Biomed. Eng..

[2] Phan T.N., Fan C.H., Yeh C.K. (2023). Application of ultrasound to enhancing stem cells associated therapies. Stem Cell Rev. Rep..

[3] Perez-Ramirez C.A., Christofk H.R. (2021). Challenges in studying stem cell metabolism. Cell Stem Cell.

[4] Romualdez-Tan M.V. (2023). Modelling in vitro gametogenesis using induced pluripotent stem cells: A review. Cell Regener..

[5] Zhuang M., Liu T., Ge D., Song K., Guan S. (2016). Preservation of osteoblasts and BM-MSCs biological properties after consecutive passages with the thermal-liftoff method. RSC Adv..

[6] Nguyen L.T.B., Odeleye A.O.O., Chui C.Y., Baudequin T., Cui Z., Ye H. (2019). Development of thermo-responsive polycaprolactone macrocarriers conjugated with poly(*N*-isopropyl acrylamide) for cell culture. Sci. Rep..

[7] Ahmadi S., Nasiri M., Pourrajab-Miandoab A., Jafari A. (2023). Temperature responsive poly(*N*-isopropylacrylamide-*co*-styrene) nanofilms for non-enzymatic cell sheet harvesting. Prog. Org. Coat..

[8] Yang L., Qiu G., Sun Y., Sun L., Fan X., Han Q., Li Z. (2024). Temperature-sensitive sensors modified with poly(*N*-isopropylacrylamide): Enhancing performance through tailored thermoresponsiveness. Molecules.

[9] Nagase K., Matsuda J., Takeuchi A., Ikemoto Y. (2023). Hydration and dehydration behaviors of poly(*N*-isopropylacrylamide)-grafted silica beads. Surf. Interfaces.

[10] Akiyama Y., Matsuyama M., Yamato M., Takeda N., Okano T. (2018). Poly(*N*-isopropylacrylamide) grafted PDMS substrate for controlling cell adhesion and detachment by dual stimulation of temperature and mechanical stress. Biomacromolecules.

[11] Kuno G., Imaizumi Y., Matsumoto A. (2022). Application of the water-insoluble, temperature-responsive block polymer poly(butyl methacrylate-*block*-*N*-isopropylacrylamide) for pluripotent stem cell culture and cell-selective detachment. J. Biosci. Bioeng..

[12] Canavan H.E., Cheng X., Graham D.J., Ratner B.D., Castner D.G. (2005). Cell sheet detachment affects the extracellular matrix: A surface science study comparing thermal liftoff, enzymatic, and mechanical methods. J. Biomed. Mater. Res. Part A.

[13] Sanzari I., Buratti E., Huang R., Tusan C.G., Dinelli F., Evans N.D., Prodromakis T., Bertoldo M. (2020). Poly(*N*-isopropylacrylamide) based thin microgel films for use in cell culture applications. Sci. Rep..

[14] Liu C., Meng M., Yang Z., Wu S., Shang L., Feng Y., Wu J., Hao K., Wang R., Zhou D. (2024). Magnetic polymer microcarriers for cell engineering applications with enzyme-free cell harvesting and rapid recovery in large-scale cell culture. Adv. Funct. Mater..

[15] Yamada N., Okano T., Sakai H., Karikusa F., Sawasaki Y., Sakurai Y. (1990). Thermo-responsive polymeric surfaces; control of attachment and detachment of cultured cells. Makromol. Chem. Rapid Commun..

[16] Yang J., Yamato M., Kohno C., Nishimoto A., Sekine H., Fukai F., Okano T. (2005). Cell sheet engineering: Recreating tissues without biodegradable scaffolds. Biomaterials.

[17] Tang Z., Akiyama Y., Yamato M., Okano T. (2010). Comb-type grafted poly(*N*-isopropylacrylamide) gel modified surfaces for rapid detachment of cell sheet. Biomaterials.

[18] Mendoza D.J., Nasiri N., Duffin R.N., Raghuwanshi V.S., Mata J., Simon G.P., Hooper J.F., Garnier G. (2024). Multifunctional graft-IPN hydrogels of cellulose nanofibers and poly(*N*-isopropyl acrylamide) via silver-promoted decarboxylative radical polymerization. Mater. Today Chem..

[19] Dzhoyashvili N.A., Thompson K., Gorelov A.V., Rochev Y.A. (2016). Film thickness determines cell growth and cell sheet detachment from spin coated poly(*N*-isopropylacrylamide) substrates. ACS Appl. Mater. Interfaces.

[20] Gilcreest V.P., Carroll W.M., Rochev Y.A., Blute I., Dawson K.A., Gorelov A.V. (2004). Thermoresponsive poly(*N*-isopropylacrylamide) copolymers: Contact angles and surface energies of polymer films. Langmuir.

[21] Lee S.B., Ha D.I., Cho S.K., Kim S.J., Lee Y.M. (2004). Temperature/pH-sensitive comb-type graft hydrogels composed of chitosan and poly(*N*-isopropylacrylamide). J. Appl. Polym. Sci..

[22] Alexander A., Ajazuddin, Khan J., Saraf S., Saraf S. (2014). Polyethylene glycol (PEG)–poly(*N*-isopropylacrylamide) (PNIPAAm) based thermosensitive injectable hydrogels for biomedical applications. Eur. J. Pharm. Biopharm..

[23] Jahn P., Karger R.K., Khalaf S.S., Hamad S., Peinkofer G., Sahito R.G.A., Pieroth S., Nitsche F., Lu J., Derichsweiler D. (2022). Engineering of cardiac microtissues by microfluidic cell encapsulation in thermoshrinking non-crosslinked PNIPAAm gels. Biofabrication.

[24] Rusen L., Dinca V., Mustaciosu C., Icriverzi M., Sima L.E., Bonciu A., Brajnicov S., Mihailescu N., Dumitrescu N., Popovici A.I., Nikitenkov N.N. (2017). Smart thermoresponsive surfaces based on pNIPAm coatings and laser method for biological applications. Modern Technologies for Creating the Thin-film Systems and Coatings.

[25] Patel N.G., Cavicchia J.P., Zhang G., Newby B.Z. (2012). Rapid cell sheet detachment using spin-coated pNIPAAm films retained on surfaces by an aminopropyltriethoxysilane network. Acta Biomater..

[26] Yang L., Fan X., Zhang J., Ju J. (2020). Preparation and characterization of thermoresponsive poly(*N*-isopropylacrylamide) for cell culture applications. Polymers.

[27] Guo Q., Ma J., Yin T., Jin H., Zheng J., Gao H. (2024). Superhydrophobic non-metallic surfaces with multiscale nano/micro-structure: Fabrication and application. Molecules.

[28] Zhuang P., Dirani A., Glinel K., Jonas A.M., Azzaroni O., Szleifer I. (2017). Temperature dependence of the swelling and surface wettability of dense polymer brushes. Polymer and Biopolymer Brushes: For Materials Science and Biotechnology.

[29] Muthiah P., Hoppe S.M., Boyle T.J., Sigmund W. (2011). Thermally tunable surface wettability of electrospun fiber mats: Polystyrene/poly(*N*-isopropylacrylamide) blended versus crosslinked poly[(*N*-isopropylacrylamide)-*co*-(methacrylic acid)]. Macromol. Rapid Commun..

[30] Liu R., Leonardis P.D., Tirelli N., Saunders B.R. (2009). Thermally-responsive surfaces comprising grafted poly(*N*-isopropylacrylamide) chains: Surface characterisation and reversible capture of dispersed polymer particles. J. Colloid Interface Sci..

[31] Kwon O.H., Kikuchi A., Yamato M., Sakurai Y., Okano T. (2000). Rapid cell sheet detachment from poly(*N*-isopropylacrylamide)-grafted porous cell culture membranes. J. Biomed. Mater. Res., Part B.

[32] Teotia A.K., Sami H., Kumar A., Zhang Z. (2015). Thermo-responsive polymers: Structure and design of smart materials. Switchable and Responsive Surfaces and Materials for Biomedical Applications.

[33] Rauch S., Eichhorn K.J., Stamm M., Uhlmann P. (2012). Spectroscopic ellipsometry of superparamagnetic nanoparticles in thin films of poly(*N*-isopropylacrylamide). J. Vac. Sci. Technol. A.

[34] Patel D.K., Lim K.T., Chauhan N.P.S. (2021). Stimuli-responsive polymers and their biomedical applications. Functionalized Polymers, Synthesis, Characterization and Applications.

[35] Yu J., Zhu L., Zhu B., Xu Y. (2011). Poly(*N*-isopropylacrylamide) grafted poly(vinylidene fluoride) copolymers for temperature-sensitive membranes. J. Membr. Sci..

[36] van der Heide P. (2011). X-ray Photoelectron Spectroscopy: An Introduction to Principles and Practices.

[37] Cheng Z., Shan H., Sun Y., Zhang L., Jiang H., Li C. (2020). Evolution mechanism of surface hydroxyl groups of silica during heat treatment. Appl. Surf. Sci..

[38] Yi B., Wang S., Hou C., Huang X., Cui J., Yao X. (2021). Dynamic siloxane materials: From molecular engineering to emerging applications. Chem. Eng. J..

[39] N’guessan H.E., White R., Leh A., Baksi A., Tadmor R., Mittal K.L. (2013). Fundamental understanding of drops wettability behavior theoretically and experimentally. Advances in Contact Angle, Wettability and Adhesion.

[40] Steiner G., Zimmerer C., Arndt K.F., Lechner M.D. (2013). Poly(N-isopropylacrylamide) Polymer Solids and Polymer Melts# Data. Polymer Solids and Polymer Melts–Definitions and Physical Properties I.

[41] Kalová J., Mareš R. (2022). Temperature dependence of the surface tension of water, including the supercooled region. Int. J. Thermophys..

[42] Zhang G., Wu C. (2001). Reentrant coil-to-globule-to-coil transition of a single linear homopolymer chain in a water/methanol mixture. Phys. Rev. Lett..

[43] Callewaert M., Grandfils C., Boulangé-Petermann L., Rouxhet P.G. (2004). Adsorption of poly(*N*-isopropylacrylamide) on glass substrata. J. Colloid Interface Sci..

[44] Rao S., Costa K.D., Morris P. (2014). Atomic force microscopy (AFM) in biomedical research. Biomedical Imaging Applications and Advances.

[45] Fan X., Gu S., Wu L., Yang L. (2020). Preparation and characterization of thermoresponsive poly(*N*-isopropylacrylamide) copolymers with enhanced hydrophilicity. e-Polymers.

[46] Furchner A., Sun G., Ketelsen H., Rappich J., Hinrichs K. (2015). Fast IR laser mapping ellipsometry for the study of functional organic thin films. Analyst.

[47] Li J., Collins R.W., Sestak M.N., Koirala P., Podraza N.J., Marsillac S., Rockett A.A., Abou-Ras D., Kirchartz T., Rau U. (2016). Spectroscopic ellipsometry. Advanced Characterization Techniques for Thin Film Solar Cells.

[48] Deng K., Wang Y., Wang L., Fan X., Wu Z., Wen X., Xie W., Wang H., Zhou Z., Chen P. (2023). Phase transition behaviors of poly(*N*-isopropylacrylamide) nanogels with different compositions induced by (−)-epigallocatechin-3-gallate and ethyl gallate. Molecules.

[49] Fan X., Lei J., Gu S., Leng X., Liu C., Yang L. (2023). Regulation of thermoresponsive PNIPAAm copolymers with enhanced hydrophilicity via NVP moieties. Mater. Rep..

[50] Himawan A., Anjani Q.K., Detamornrat U., Vora L.K., Permana A.D., Ghanma R., Naser Y., Rahmawanty D., Scott C.J., Donnelly R.F. (2023). Multifunctional low temperature-cured PVA/PVP/citric acid-based hydrogel forming microarray patches: Physicochemical characteristics and hydrophilic drug interaction. Eur. Polym. J..

[51] Bueno V.B., Bentini R., Catalani L.H., Petri D.F.S. (2013). Synthesis and swelling behavior of xanthan-based hydrogels. Carbohydr. Polym..

[52] Gong L., Liu D., Fan T., Qin J., Li J., Zhang Q., Wu Z., Fan Z., Liu Q. (2020). Surface modification of PTLG terpolymer using PNIPAAm and its cell adhesion/detachment behavior. Macromol. Chem. Phys..

[53] Lian J., Xu H., Duan S., Ding X., Hu Y., Zhao N., Ding X., Xu F.J. (2020). Tunable adhesion of different cell types modulated by thermoresponsive polymer brush thickness. Biomacromolecules.

[54] Cooperstein M.A., Canavan H.E. (2010). Biological cell detachment from poly(*N*-isopropyl acrylamide) and its applications. Langmuir.

[55] Nitschke M., Gramm S., Götze T., Valtink M., Drichel J., Voit B., Engelmann K., Werner C. (2007). Thermo-responsive poly(NiPAAm-*co*-DEGMA) substrates for gentle harvest of human corneal endothelial cell sheets. J. Biomed. Mater. Res. Part A.

[56] Ernst O., Lieske A., Jäger M., Lankenau A., Duschl C. (2007). Control of cell detachment in a microfluidic device using a thermo-responsive copolymer on a gold substrate. Lab Chip.

[57] Canavan H.E., Cheng X., Graham D.J., Ratner B.D., Castner D.G. (2005). Surface characterization of the extracellular matrix remaining after cell detachment from a thermoresponsive polymer. Langmuir.

[58] Akiyama Y., Kushida A., Yamato M., Kikuchi A., Okano T. (2007). Surface characterization of poly(*N*-isopropyl acrylamide) grafted tissue culture polystyrene by electron beam irradiation, using atomic force microscopy, and X-ray photoelectron spectroscopy. J. Nanosci. Nanotechnol..

[59] Kanai N., Yamato M., Okano T., Orlando G., Lerut J., Soker S., Stratta R.J. (2014). Principles of cell sheet technology. Regenerative Medicine Applications in Organ Transplantation.

[60] Lefebvre J., Galli F., Bianchi C.L., Patience G.S., Boffito D.C. (2019). Experimental methods in chemical engineering: X-ray photoelectron spectroscopy-XPS. Can. J. Chem. Eng..

[61] Bi Y., Chen L., Duan C., Zhao Z. (2024). OER performance of Ce-CoFe-P@CC nanocomposite catalytic system. J. Petrochem. Univ..

[62] Iucci G., Battocchio C., Dettin M., Ghezzo F., Polzonetti G. (2010). An XPS study on the covalent immobilization of adhesion peptides on a glass surface. Solid State Sci..

[63] Ahmadi S., Nasiri M., Pourrajab-Miandoab A., Abbasi F., Hassanpour F., Sabzi E. (2022). Photo- and thermo-responsive extracellular matrix mimicking nano-coatings prepared from poly(*N*-isopropylacrylamide)-spiropyran copolymer for effective cell sheet harvesting. Prog. Org. Coat..

[64] Zhang Z., Cao X., Zhao X., Withers S.B., Holt C.M., Lewis A.L., Lu J.R. (2006). Controlled delivery of antisense oligodeoxynucleotide from cationically modified phosphorycholine polymer films. Biomacromolecules.

