# Supplementary material for: Engineering Thermoresponsive Poly(N-isopropylacrylamide)-Based Films with Enhanced Stability and Reusability for Efficient Bone Marrow Mesenchymal Stem Cell Culture and Harvesting
Source: Molecules. 2024 Sep 21;29(18):4481. doi: 10.3390/molecules29184481 (PMC11435103; doi:10.3390/molecules29184481)
Supplement: Supplementary file 1 [file molecules-29-04481-s001.zip › molecules-3179514-supplementary.pdf]

Table S1. Performance stability of P(NIPAM-*co*-MPS-*co*-HPMA) copolymer films (1 mg·mL^{-1}) after different treatment.

Treatment	Before treatment		After treatment	
	Contact angle (°)	Film thickness (nm)	Contact angle (°)	Film thickness (nm)
Stored in sealed, dry, and sterile environments at 4 °C	55.1±0.23	3.0±0.11	54.6±0.31	3.0±0.16
Stored in sealed, dry, and sterile environments at 20 °C	55.3±0.46	3.1±0.19	55.0±0.37	3.1±0.10
Stored in sealed, dry, and sterile environments at 37 °C	54.9±0.28	3.0±0.13	55.3±0.17	3.0±0.22
Immersed in ultrapure water and underwent three rounds of alternating heating and cooling	55.2±0.41	3.0±0.10	53.6±0.28	2.9±0.27
